# The Birth of the Copenhagen School: Personal Recollections at the EMBO Workshop on Bacterial Growth Physiology, 2022

**DOI:** 10.3390/life13122235

**Published:** 2023-11-21

**Authors:** Moselio Schaechter

**Affiliations:** Division of Biological Sciences, University of California, San Diego, CA 92093, USA; elios179@gmail.com

The future of bacterial growth physiology is shining more brightly than ever. It is profiting from our increased ability to study individual bacteria, from advances in different forms of microscopy, and from the increased use of computational modeling. Bacterial physiology is merging with systems biology (a term that is perhaps less in vogue) and is now increasingly influenced by physical chemistry.

Our knowledge of bacteria continues to grow as we learn more and more about the interactions in living cells between and among micro- and macro- molecules, the happenings during the cell cycle, the variability of what were once thought to be homogeneous populations, and other such alluring subjects. These advances are due in no small measure to the return to biology of physicists, who introduce new ways of thinking via new technologies and new approaches to modeling and simulation.

That being said, the old, fundamental questions remain. Why do bacteria such as *Escherichia coli* have so many regulatory mechanisms that overlap? What are the underlying rules? How is it possible that in bacteria (as opposed to eukaryotes), the major macromolecular factories can, to some extent, operate independently of one another? In the broadest terms, questions related to morphology, chromosome replication, segregation, and cell division—and the couplings between them—are being posed in new ways, for example, in terms of polymer physics. This Special Issue follows the EMBO workshop in Israel “Bacterial cell biophysics: DNA replication, growth, division, size and shape” (2022) at which The Charles E. Helmstetter Prize for Groundbreaking Research in Bacterial Cell Cycle Physiology was inaugurated. I am a recipient of this prize, and am certain that had I been at the meeting, I would have found that the number of fascinating topics is increasing enormously, and that excitement in the field continues to mount.

**The Birth of “The Copenhagen School”**

I should know. I was there. But first, I arrived in Copenhagen in 1956 after a two-year stint at the Walter Reed Army Research Institute. Why Copenhagen and why Ole Maaløe’s lab? A couple of good reasons. My wife at the time, Barbara, had taken a college course in Scandinavian Civilization and fell in love with both the topic and the teacher. Also, at the time, I found my barracks-mate, the soon-to-become famous phage geneticist Allan Campbell, sprawled on his Army bunk, reading a paper by Lark and Maaløe on synchronizing bacterial growth. He proclaimed aloud that ”everything that has been done has to be done over”. That was good enough for me although, uncharacteristically, Allan was wrong on this one. The reason, as was found out later in Ole’s own lab, was that the temperature shifts used by Lark and Maaløe to induce synchronous divisions created artefacts. But I did not know this at the time, so I went there undeterred.

Ole had just returned from a year at Caltech, where he had been immersed in “Delbrückian” molecular biology. There, he published several papers on phage development with Jim Watson and Gunther Stent. Before that, Ole had attended medical school in Copenhagen. Upon his return to Denmark, he took a job in the department for the standardization of sera and vaccines of the State Serum Institute. As it turned out, this was a rather undemanding position. The nominal work was done almost entirely by Ole’s assistant, the highly gifted Jens Ole Rostock. This left Ole with plenty of time to develop his interest in molecular biology. In time, Ole’s lab (soon at the University of Copenhagen) became prominent, a place at which Americans and others could carry out interesting studies in molecular biology. Eventually, this led to the creation of the Copenhagen School of Bacterial Growth Physiology.

A few words about Ole as a person. In general terms, he was very much a Dane: somewhat reserved, yet sensitive to the needs for the well-being of his fellow men. I believe that this is indeed common in Denmark, a country which, by the way, rates No. 2 in the world in personal happiness. Ole had a special interest in oriental, read a lot about them, and collected mandalas. He was seldom seen without a cigar in his mouth which he temporarily removed, reluctantly, when mouth pipetting.

The birth of the Copenhagen School can be traced to the following modest event: Ole was looking for a way to detect synchronous growth faster than by counting cells via colony plate counts (which takes about a day). He had just acquired a new gadget, a Zeiss PMQII spectrophotometer. He imagined that with such a highly sensitive apparatus, he could detect small jumps in the optical density of a culture undergoing synchronous growth. But when we “shifted up” a culture in minimal medium by adding a rich medium to it, we saw no special jumps, just a steady increase in the growth rate. Abandoning his thoughts of synchronous division, Ole proposed that the speed at which bacteria grow may dictate their size (at the time, such thoughts were relegated to the messy corners of bacteriology). He had his post-doctoral students, Niels Ole Kjeldgaard and I, to test this notion by growing the bacterium *Salmonella typhimurium* in media of different richnesses, from a salt mixture with a single carbon source to a very rich broth. (By the way, with Ole, Jens Ole, and Niels Ole on board, I thought I should change my name to Elio Ole).

We were blessed by the Serum Institute, which had a most accommodating media kitchen. Whatever media we ordered, simple or complex, we received within 24 h. So, day after day, we grew our bacteria in a variety of different media. We expressed cell mass, ergo, cell size, as the ratio of the optical density of the culture vs. the number of cells (obtained via colony counts after plating). Sure enough, we soon found that Ole was correct: the faster the growth rate afforded by the medium, the larger the cells, in a direct relationship. Having measured the cells’ content of nucleic acids, we could postulate that at a given temperature, the unit rate of macromolecular synthesis was constant in all media, something that was later demonstrated via direct measurements. Although this point was hinted at by previous work, ours was rendered more believable by our systematic approach and the detailed care with which we made the measurements. Additionally, Ole found convincing ways of making this business sound important.

I left Ole’s lab in 1958 for my first academic job (at the University of Florida in Gainesville). Shortly thereafter, Ole was named the first professor of Microbiology at the University of Copenhagen. With the help of Jens Ole Rostock, he built up an exceptional research institution that attracted a large number of first-rate local and international investigators. Ole received many recognitions as a leading light in Danish science. A street in Copenhagen was named after him (Figure 1, from Anderson KB et al. [1]). He died in 1988.

**My “After Copenhagen”**

So, what did I do next? With a lab of my own and total freedom of action, should I follow up on the work in Copenhagen or try something different? I first tried a bit of both, but soon settled on the latter. Influenced by the developments in eukaryotic cell biology depending on cell fractionation techniques, I wanted to see what I would find when I opened up my bacterial cells. The conventional way to do this involves rather punitive methods, such as mangling the cells via sonication and grinding them with alumina. A gentle way to do it is to convert the bacteria into spheroplasts, cell-wall-less entities that can be lysed using a whiff of detergent. Sure enough, when we did this, we found that the ribosomes of the cell were linked together on nascent messenger RNA molecules in what soon became known as polyribosomes or polysomes. We visualized them using two methods, sucrose gradients and an analytical ultracentrifuge. This was a venerable and lumbering machine known as the Beckman Model E which, at that time, was the pride of any department that owned one (go see if you can find one anywhere now).

An unexpected event led us to study the attachment of DNA to the cell membrane. To lyse our spheroplasts, we decided to try a new detergent, sodium lauryl sarcosinate. To our surprise, after we centrifuged the gradients, we saw a thin white band about halfway down the tubes. This turned out to consist of the insoluble crystals of magnesium (present in our buffer) lauryl sarcosinate. Somehow, instead of abandoning the experiment, we analyzed the contents of the tubes and, to our surprise, found that all of the cells’ DNA was in the white band, which was nicknamed the “M-band”. About 30% of the cells’ membranes were in the white band as well, but purified DNA was not. We reckoned that the DNA we found in this M-band was attached to the membrane. Probing further into it, we determined that the DNA is attached to the membrane at some 20 attachment sites.

At about that time, I closed my lab and moved from Boston to San Diego, where I have since been using social media to spread the microbiological word via a blog entitled “Small Things Considered” at http://schaechter.asmblog.org/ (accessed on 6 September 2023) or http://smallthingsconsidered.us (accessed on 6 September 2023) and a podcast, “This Week in Microbiology” (TWiM), at https://podcasts.google.com/search/%22This%20Week%20in%20Microbiology%22?hl=en-IL (accessed on 6 September 2023). A few selected publications relevant to this story are cited below [2,3,4,5,6,7,8,9,10,11,12,13,14,15,16,17].

## Figures and Tables

**Figure 1:**
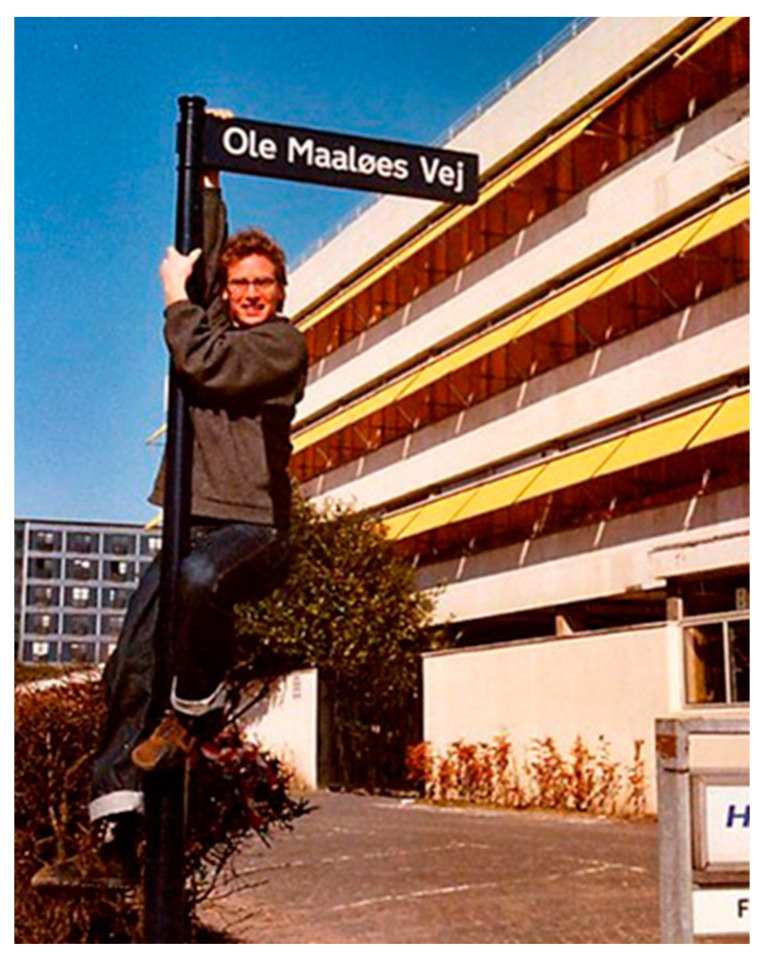
Street sign (Ole Maaløe s’ Way) and Ole’s grandson.
